# Unravelling The Enigma of Pseudocyesis in Females

**DOI:** 10.1192/j.eurpsy.2026.11958

**Published:** 2026-07-31

**Authors:** K. Anurag, L. Dash

**Affiliations:** Psychiatry, Ims & Sum Hospital Campus Ii, Bhubaneshwar, India

## Abstract

**Introduction:**

Pseudocyesis, or false pregnancy, is a rare psychosomatic disorder where a woman develops a firm belief of being pregnant, accompanied by physical signs mimicking gestation, despite the absence of actual pregnancy. It is more prevalent among women aged 20–40 years, particularly in rural areas of developing countries, and is often associated with low socioeconomic status and strong sociocultural expectations of motherhood. It is a psych neuroendocrine condition where emotional factors disrupt hormonal and neural regulation involving HPO axis, limbic system and dopamine transmission. According to ICD-11, it aligns with 6B42: Somatic Symptom Disorder, while DSM-5 classifies it under Somatic Symptom and Related Disorders.

**Objectives:**

assessing clinical features, management challenges in pseudocyesis

**Methods:**

This case series includes six patients diagnosed with pseudocyesis over a span of five years. These women initially sought care in departments such as Obstetrics and Gynecology and Surgery, and were subsequently referred to Psychiatry OPD at IMS & SUM Hospital CAMPUS II. All cases presented with signs suggestive of pregnancy but had negative β-hCG and normal pelvic ultrasound findings. Comprehensive psychiatric evaluations were conducted, including application of HAM-A, HAMD, and Y-BOCS to screen for anxiety, depression, and obsessive-compulsive traits, along with personality assessments to identify Cluster C characteristics tailored to each patient’s psychosocial context.

**Results:**

Patients presented with typical symptoms such as amenorrhea, abdominal distension, breast changes, weight gain, and nausea/vomiting. One patient also reported perceived fetal movements. Despite negative pregnancy tests and imaging, each patient maintained a strong belief in being pregnant. Psychological stressors, including family pressure, infertility concerns, trauma, and mood symptoms, were common. Symptoms developed insidiously over 1–4 months. None of the cases involved conscious symptom fabrication, ruling out malingering or factitious disorder. Psychological interventions led to significant improvement in all cases. Pharmacotherapy was reserved only for comorbid psychiatric conditions and not required for somatic complaints.

**Image 1:**
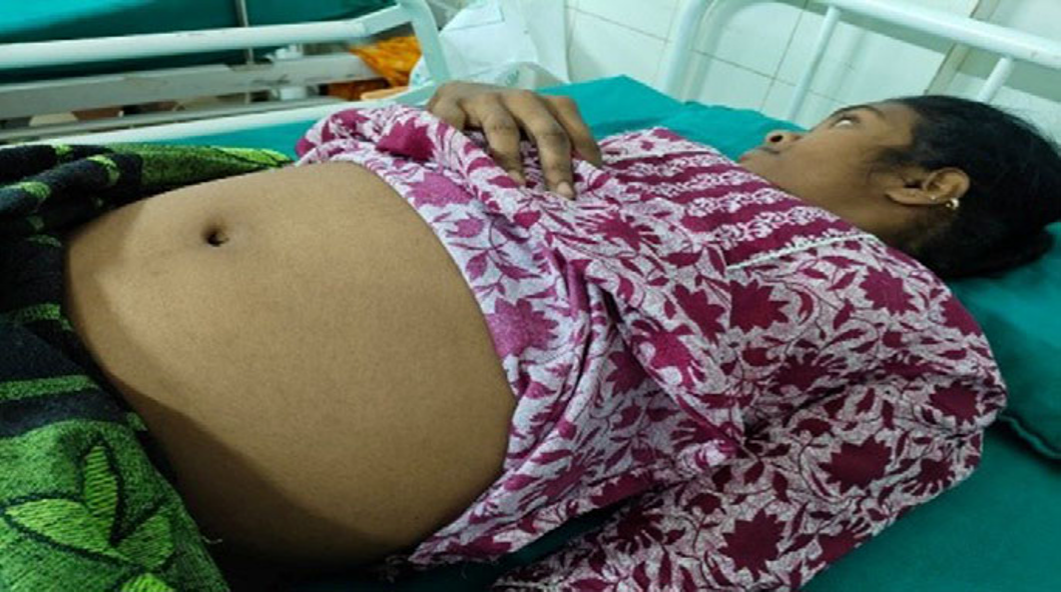

**Conclusions:**

In management of pseudocyesis, medications such as SSRI proved to be effective, along with psychotherapy and psychoeducation. It is also essential to rule out psychotic symptoms along with depression and anxiety to ensure accurate diagnosis and appropriate anti-psychotic management. Challenges include strong patient belief, emotional sensitivity, cultural stigma, and resistance to psychiatric care. Early intervention has shown positive outcomes, but many cases go unrecognized due to limited awareness. Proper research into this disorder is essential to develop evidence based guidelines for the effective future management of this disorder.

**Disclosure of Interest:**

None Declared

